# Factors Associated With Experiences of Fear, Anxiety, Depression, and Changes in Sleep Pattern During the COVID-19 Pandemic Among Adults in Nigeria: A Cross-Sectional Study

**DOI:** 10.3389/fpubh.2022.779498

**Published:** 2022-03-02

**Authors:** Morenike Oluwatoyin Folayan, Olanrewaju Ibigbami, Brandon Brown, Maha El Tantawi, Nourhan M. Aly, Oliver C. Ezechi, Giuliana Florencia Abeldaño, Eshrat Ara, Martin Amogre Ayanore, Passent Ellakany, Balgis Gaffar, Nuraldeen Maher Al-Khanati, Ifeoma Idigbe, Mohammed Jafer, Abeedha Tu-Allah Khan, Zumama Khalid, Folake Barakat Lawal, Joanne Lusher, Ntombifuthi P. Nzimande, Bamidele Olubukola Popoola, Mir Faeq Ali Quadri, Mark Roque, Ala'a B. Al-Tammemi, Muhammad Abrar Yousaf, Jorma I. Virtanen, Roberto Ariel Abeldaño Zuñiga, Nicaise Ndembi, John N. Nkengasong, Annie Lu Nguyen

**Affiliations:** ^1^Mental Health and Wellness Study Group, Ile-Ife, Nigeria; ^2^Department of Child Dental Health, Obafemi Awolowo University, Ile-Ife, Nigeria; ^3^Department of Mental Health, Obafemi Awolowo University, Ile-Ife, Nigeria; ^4^Department of Social Medicine, Population and Public Health, Riverside School of Medicine, University of California, Riverside, Riverside, CA, United States; ^5^Department of Pediatric Dentistry and Dental Public Health, Faculty of Dentistry, Alexandria University, Alexandria, Egypt; ^6^Department of Clinical Sciences, Nigerian Institute of Medical Research, Lagos, Nigeria; ^7^School of Medicine, University of Sierra Sur, Oaxaca, Mexico; ^8^Government College for Women, Srinagar, India; ^9^Department of Health Policy Planning and Management, School of Public Health, University of Health and Allied Sciences, Ho, Ghana; ^10^Department of Substitutive Dental Sciences, Imam Abdulrahman Bin Faisal University, Dammam, Saudi Arabia; ^11^Department of Preventive Dental Sciences, College of Dentistry, Imam Abdulrahman bin Faisal University, Dammam, Saudi Arabia; ^12^Department of Oral and Maxillofacial Surgery, Faculty of Dentistry, Syrian Private University, Damascus, Syria; ^13^Department of Preventive Dental Sciences, Saudi Arabia Department of Health Promotion, Faculty of Health, Medicine and Life Sciences, Jazan University, Maastricht University, Maastricht, Netherlands; ^14^Department of Health Sciences, University of Genova, Genova, Italy; ^15^Department of Periodontology and Community Dentistry, University of Ibadan and University College Hospital, Ibadan, Nigeria; ^16^Director of People and Member of the Provost's Group, Regent's University, London, United Kingdom; ^17^Department of Economic and Social Geography, University of Szeged, Szeged, Hungary; ^18^Department of Child Oral Health, University of Ibadan, Ibadan, Nigeria; ^19^Department of Preventive Dental Sciences, Division of Dental Public Health, College of Dentistry, Jazan University, Jizan, Saudi Arabia; ^20^Department of Maternity and Childhood Nursing, College of Nursing, Taibah University, Medina, Saudi Arabia; ^21^Migration Health Division, International Organization for Migration (IOM), The UN Migration Agency, Amman, Jordan; ^22^Institute of Zoology, University of the Punjab, Lahore, Pakistan; ^23^Faculty of Medicine, University of Turku, Turku, Finland; ^24^Postgraduate Department, University of Sierra Sur, Oaxaca, Mexico; ^25^Africa Centres for Disese Control and Preventon, African Union Commission, Addis Ababa, Ethiopia; ^26^Department of Family Medicine, Keck School of Medicine, University of Southern California, Los Angeles, CA, United States

**Keywords:** SARS-CoV-2, mental health, HIV, COVID-19, Nigeria, mental distress

## Abstract

**Background:**

Multiple facets of the pandemic can be a source of fear, depression, anxiety and can cause changes in sleep patterns. The aim of this study was to identify health profiles and the COVID-19 pandemic related factors associated with fear, depression, anxiety and changes in sleep pattern in adults in Nigeria.

**Methods:**

The data for this analysis was extracted from a cross-sectional online survey that collected information about mental health and well-ness from a convenience sample of adults 18 years and above resident in Nigeria from July to December 2020. Study participants were asked to complete an anonymous, closed-ended online questionnaire that solicited information on sociodemographic profile, health profiles (high, moderate and low COVID-19 infection risk profile) including HIV status, COVID-19 status, and self-reported experiences of fear, anxiety, depression and changes in sleep patterns.

**Results:**

In total, 4,439 participants with mean age of 38.3 (±11.6) years responded to the survey. Factors associated with higher odds of having COVID-19 related fear were health risk (*p* < 0.05); living with HIV (AOR: 3.88; 95% CI: 3.22–4.69); having COVID-19 symptoms but not tested (AOR: 1.61; 95% CI: 1.30–1.99); having a friend who tested positive to COVID-19 (AOR: 1.28; 95% CI: 1.07–1.53) and knowing someone who died from COVID-19 (AOR: 1.43; 95% CI: 1.24–1.65). The odds of feeling anxious was significantly higher for those with moderate or low health risk profile (*p* < 0.05); living with HIV (AOR: 1.64; 95% CI: 1.32–2.04); had a friend who tested positive for COVID-19 (AOR: 1.35; 95% CI: 1.08–1.68) or knew someone who died from COVID-19 (AOR: 1.53; 95% CI: 1.28–1.84). The odds of feeling depressed was significantly higher for those with health risk profile (*p* < 0.05); living with HIV (AOR: 2.49; 95% CI: 1.89–3.28); and respondents who had COVID-19 symptoms but had not taken a test (AOR: 1.41; 95% CI: 1.02–1.94). Factors associated with higher odds of having sleep pattern changes were having moderate and low health risk profiles (*p* < 0.05).

**Conclusion:**

The study findings suggest that the pandemic may cause fear, anxiety, depression and changes in sleep patterns differently for people with different health profile, HIV status and COVID-19 status.

## Introduction

For many individuals, the COVID-19 pandemic has been a source of fear, depression, and anxiety; all of which can lead to changes in sleep quality and patterns. Multiple facets and characteristics of the pandemic can be attributed to these outcomes. Concerns about mortality and morbidity associated with the COVID-19, scarcity of financial resources, and uncertainty about time of recovery from associated financial hardships are partly to blame (1). Patients with COVID-19 also fear abandonment, feelings of isolation and psychological sufferings (1). Some may fear infecting friends and family members, otherwise known as contamination fear (2–4). The fear of the unknown appears to be a core component of anxiety that accompanies situations that are unpredictable and uncontrollable (5, 6). Fear of these threats is often learned, irrespective of the probability of its occurrence, and results from the inability to tolerate uncertainty (7). The intolerance of uncertainty is also related to depressive symptomatology, and the fear of COVID-19 may explain part of the relation (8).

The COVID-19 pandemic is associated with up to a seven times higher prevalence of depression (9) and over 25% mental deterioration in some populations (10, 11). Persons with prior history of mental health disorders had higher rates of depression during the pandemic (12). Depressive symptoms were associated with testing positive for COVID-19 or having COVID-19 symptoms, exposure to social media, poor social support, unemployment, uncertainty about the future of jobs, and careers and economic crisis, especially for students (9). As with fear, depression is associated with anxiety (13, 14). The prevalence of anxiety during the COVID-19 pandemic is higher than 30% (15); and anxiety is higher in people with poor health (16). Anxiety disorder may lead to dysfunctional arousal that in turn results in persistent sleep-wake difficulties such as insomnia and hypersomnia (17, 18). Sleep disturbance is also a diagnostic symptom for generalized anxiety disorder (19), with young people being the worse-affected (20).

Though the prevalence of sleep problems, fear, anxiety and depression increased during the pandemic (21, 22), the impact may, however, differ between populations (23, 24). Fear, anxiety, depression and sleep disorder may be lower in the general population than it is in populations living with co-morbidities. Understanding the association between negative emotions and sleep pattern during the COVID-19 pandemic is important. However, research in this field is scarce (20). We hypothesize that respondent's COVID-19 related status would be associated with the experience of fear, depression, anxiety and changes in sleep pattern during the pandemic; that more people living with HIV will experience fear, anxiety, depression and sleep disorder; and that more people with fear, anxiety, depression and sleep disorder will use COVID-19 preventive measures.

The consolidation of contextual fear, depression, anxiety and avoidance of the shock evoke negative emotions and trigger alterations in sleep characteristics (25). Despite this, there is a little known about the aspects of the pandemic crisis that trigger negative emotions. One of the aims of this study was to identify COVID-19 pandemic related factors such as COVID-19 test positivity status, history of COVID-19 symptoms, and contact/relation with persons who have COVID-19, and their association with fear, depression, anxiety, and changes in sleep pattern. We also identified the association between fear, depression, anxiety, and changes in sleep pattern and the COVID-19 status. Finally, we determined if living with HIV was associated with the experience of fear, depression, anxiety, and changes in sleep pattern.

## Methods

### Study Design and Study Participants

This was a sub-analysis data from an international cross-sectional study on the impact of COVID-19 on the mental health and well-ness of adults using an online multi-country survey. Data were collected from a convenient sample of adults 18 years and above from July to December 2020. The study methodology had been reported in detail in prior studies (26, 27).

### Study Instrument

The survey used a questionnaire, which was initially developed for a study that targeted a specific population in the United States and was consequently adapted and validated for use by a global audience (28). The questionnaire underwent four iterative processes content validation. The overall content validity index of the survey was 0.83. The responses collected for content validation were excluded from the final analysis. The study was approved by the Human Research Ethics Committee at the Institute of Public Health of the Obafemi Awolowo University Ile-Ife, Nigeria (HREC No: IPHOAU/12/1557). Participants received no incentive for taking part in the study.

### Recruitment of the Study Participants

A call for collaboration for this study was made on Research gate. The 45 collaborators engaged through the public call were required to distribute their unique survey links to networks within and outside their countries and communities to ensure maximum representation and geographic spread. There were none data collectors recruited from Nigeria. The study participants were recruited through respondent-driven sampling. These links were posted on social media groups (Facebook, Twitter, and Instagram) and sent via WhatsApp or email to eligible participants in each collaborators' networks. The study participants were further asked to disseminate the links to those in their own networks using snowball sampling to facilitate further recruitment. The survey link was also posted on social media groups (Facebook, Twitter, and Instagram, WhatsApp) and network email lists.

### Data Collection

Study participants were asked to complete an anonymous, closed-ended questionnaire to learn about how the COVID-19 pandemic has affected the people's mental health and psychological wellbeing. The questionnaire also enquired about respondents' sociodemographic profile, health profile, and various aspects of pandemic-related stress. The questionnaire was preceded by a brief introduction explaining the purpose of the study, and assuring participants of their voluntary participation, and confidentiality of their data. The questionnaire took an average of 11 min to complete and was administered in English. Each participant could only complete a single questionnaire through IP address restrictions, though they could edit their answers freely until they chose to submit. For the current analyses, we included only respondents who self-reported as residing in Nigeria. We also identified and removed survey responses that were completed below 7 min—the minimum time for filling the questionnaire by people familiar with the questionnaire in the pilot stage (*n* = 77); and those with incomplete data on fear, anxiety, depression and sleep disorder (*n* = 32).

### Explanatory Variables

#### Sociodemographic Variables

The section on sociodemographic profile had questions on country of residence, age (in years), sex at birth, highest level of education attained (none, primary, secondary and tertiary) and employment status (retired, student, employed, and unemployed).

#### Health Profile

The section on health profile required respondents to select any of the 23 medical conditions listed that they experienced in addition to other health conditions not listed. These medical conditions put individuals at high risk for severe COVID-19 disease (pneumonia, diabetes, cancer, heart condition), those that might put people at moderate risk for severe COVID-19 disease (hepatitis, hypertension, neurological problems, neuropathy, respiratory problems, stroke, depression) and those conditions associated with low risk for severe COVID-19 disease (herpes, shingles and other sexually transmitted infections, dermatologic problems, migraines, arthritis, broken bones, hearing loss and vision loss) (29). As part of the list, participants were also asked about their HIV status. A tick on a checkbox on the list of health conditions was an indication that the individual had the health condition. All respondents were categorized as either having the health condition (indicated by a tick of the checkbox) or not having the health condition (indicated by not ticking the checkbox).

#### COVID-19 Status

Respondents were asked if they had tested positive for COVID-19, had COVID-19 symptoms but did not test, had a close friend who tested positive for COVID-19, or knew someone who died from COVID-19. Response choices for these items were “yes” or “no”.

### Outcome Variables

#### Fear, Anxiety and Depression

Respondents were asked to indicate if they had experienced fear, anxiety and depression during the pandemic by checking a response box. The questions were adapted from the Pandemic Stress Index (30).

#### Changes in Sleep Pattern

Respondents were asked to indicate if they had experienced changes in sleep patterns (sleeping more, sleeping less, or no changes) during the pandemic. Each respondent was required to check a response box that indicated if they had experienced any of these conditions. The questions were adapted from the Pandemic Stress Index (30). The responses were dichotomised to change (sleeping more, sleeping less) and no change in sleep pattern.

### Data Analysis

Data were downloaded from Survey Monkey® as SPSS file version 23.0 (IBM Corp., Armonk, N.Y., USA), cleaned and prepared for analysis. T- test and chi square tests were used to assess the relationship between COVID-19 status (testing positive, suspected but not tested, friend testing positive and knowing someone who died of COVID-19) on one hand, and health profile, HIV status, fear, anxiety, depression, and changes in sleep pattern on the other hand. Also, the associations between the explanatory variables and the outcome variables were determined by conducting logistic regression analysis using four models: one for each outcome variable. The covariates for the study were the sociodemographic profile (age, sex, educational level, and employment status). Adjusted odds ratios, 95% confidence intervals (CIs) and *p*-values were calculated. Significance was set at 5%.

## Results

The mean age of the 4,439 respondents living in Nigeria was 38.3 years (SD = 11.6) ranging from 18 years to 85 years. Table 1 highlights the demographic profile of respondents. More than half of the respondents were females (53.2%), the majority had college/university education (80.9%) and were employed (70.5%). Also, 110 (2.5%) respondents tested positive for COVID-19, 466 (10.5%) had COVID-19 symptoms but did not take a test, 715 (16.1%) had a friend who had tested positive to COVID-19, and 1,367 (30.8%) knew someone who died of COVID-19. The majority (52.9%) expressed fear in response to the pandemic—fear of getting infected (50.7%) or fear of infecting someone (11.3%). Moreover, 746 (16.8%) felt anxious, 389 (8.8%) felt depressed and 1,007 (22.7%) experienced changes in their sleep pattern.

**Table 1:** Factors associated with COVID-19 status by adults in Nigeria (*N* = 4,439).

**Variables**	**Total** ***N* = 4,439 *n* (%)**	**COVID-19 positive**			**Had COVID-19 symptoms but no test**			**Friend tested positive to COVID-19**			**Knew someone who died of COVID-19**		
		**No** ***N* = 4,329** ***n* (%)**	**Yes** ***N* = 110** ***n* (%)**	***P*-value**	**No** ***N* = 3,973** ***n* (%)**	**Yes** ***N* = 466** ***n* (%)**	***P*-value**	**No** ***N* = 3,724** ***n* (%)**	**Yes** ***N* = 715** ***n* (%)**	***P*-value**	**No** ***N* = 3,072** ***n* (%)**	**Yes** ***N* = 1,367** ***n* (%)**	***P*-value**
Age Mean (SD) in years	38.30 (11.63)	38.31 (11.59)	39.58 (12.72)	0.256	38.77 (11.71)	34.64 (10.15)	<0.001	38.11 (11.82)	39.55 (10.45)	0.002	37.16 (11.38)	40.99 (11.72)	<0.001
**Sex**													
Male	2,076 (46.8)	2,020 (97.3)	56 (2.7)	0.386	1,829 (88.1)	247 (11.9)	0.004	1,716 (82.7)	360 (17.3)	0.036	1,358 (65.4)	718 (34.6)	<0.001
Female	2,363 (53.2)	2,309 (97.7)	54 (2.3)		2,144 (90.7)	219 (9.3)		2,008 (85.0)	355 (15.0)		1,714 (72.5)	649 (27.5)	
**Level of education**													
No formal education	48 (1.1)	46 (95.8)	2 (4.2)	0.689	42 (87.5)	6 (12.5)	0.764	43 (89.6)	5 (10.4)	<0.001	37 (77.1)	11 (22.9)	<0.001
Primary	84 (1.9)	82 (97.6)	2 (2.4)		76 (90.5)	8 (9.5)		77 (91.7)	7 (8.3)		66 (78.6)	18 (21.4)	
Secondary	715 (16.1)	701 (98.0)	14 (2.0)		633 (88.5)	82 (11.5)		664 (92.9)	51 (7.1)		604 (84.5)	111 (15.5)	
College/university	3,592 (80.9)	3,500 (97.4)	92 (2.6)		3,222 (89.7)	370 (10.3)		2,940 (81.8)	652 (18.2)		2,365 (65.8)	1,227 (34.2)	
**Employment status**													
**Current status**													
Retired	122 (2.7)	118 (96.7)	4 (3.3)	0.163	117 (95.9)	5 (4.1)	0.002	112 (91.8)	10 (8.2)	<0.001	77 (63.1)	45 (36.9)	<0.001
Student	495 (11.2)	489 (98.8)	6 (1.2)		425 (85.9)	70 (14.1)		456 (92.1)	39 (7.9)		398 (80.4)	97 (19.6)	
Employed	3,131 (70.5)	3,045 (97.3)	86 (2.7)		2,822 (90.1)	309 (9.9)		2,517 (80.4)	614 (19.6)		2,053 (65.6)	1,078 (34.4)	
Unemployed	691 (15.6)	677 (98.0)	14 (2.0)		609 (88.1)	82 (11.9)		639 (92.5)	52 (7.5)		544 (78.7)	147 (21.3)	
**Medical health profile**													
**High risk**													
No	4,272 (96.2)	4,171 (97.6)	101 (2.4)	0.014	3,828 (89.6)	444 (10.4)	0.250	3,593 (84.1)	679 (15.9)	0.051	2,977 (69.7)	1,295 (30.3)	<0.001
Yes	167 (3.8)	158 (94.6)	9 (5.4)		145 (86.8)	22 (13.2)		131 (78.4)	36 (21.6)		95 (56.9)	72 (43.1)	
**Moderate risk**													
No	3,742 (84.3)	3,657 (97.7)	85 (2.3)	0.040	3,376 (90.2)	366 (9.8)	<0.001	3,166 (84.6)	576 (15.4)	0.003	2,647 (70.7)	1,095 (29.3)	<0.001
Yes	697 (15.7)	672 (96.4)	25 (3.6)		597 (85.7)	100 (14.3)		558 (80.1)	139 (19.9)		425 (61.0)	272 (39.0)	
**Low risk**													
No	3,986 (89.8)	3,895 (97.7)	91 (2.3)	0.013	3,596 (90.2)	390 (9.8)	<0.001	3,359 (84.3)	627 (15.7)	0.043	2,797 (70.2)	1,189 (29.8)	<0.001
Yes	453 (10.2)	434 (95.8)	19 (4.2)		377 (83.2)	76 (16.8)		365 (80.6)	88 (19.4)		275 (60.7)	178 (39.3)	
**HIV Status**													
Living with HIV	912 (20.5)	904 (99.1)	8 (0.9)	<0.001	819 (89.8)	93 (10.2)	0.740	830 (91.0)	82 (9.0)	<0.001	740 (81.1)	172 (18.9)	<0.001
Not living with HIV	3,527 (79.5)	3,425 (97.1)	102 (2.9)		3,154 (89.4)	373 (10.6)		2,894 (82.1)	633 (17.9)		2,332 (66.1)	1,195 (33.9)	
**COVID 19 related fear**													
**Fear of getting infected**													
No	2,189 (49.3)	2,108 (96.3)	81 (3.7)	<0.001	1,998 (91.3)	191 (8.7)	<0.001	1,858 (84.9)	331 (15.1)	0.078	1,565 (71.5)	624 (28.5)	0.001
Yes	2,250 (50.7)	2,221 (98.7)	29 (1.3)		1,975 (87.8)	275 (\12.2)		1,866 (82.9)	384 (17.1)		1,507 (67.0)	743 (33.0)	
**Fear of infecting someone else**													
No	3,936 (88.7)	3,857 (98.0)	79 (2.0)	<0.001	3,599 (91.4)	337 (8.6)	<0.001	3,379 (85.8)	557 (14.2)	<0.001	2,794 (71.0)	1,142 (29.0)	<0.001
Yes	503 (11.3)	472 (93.8)	31 (6.2)		374 (74.4)	129 (25.6)		345 (68.6)	158 (31.4)		278 (55.3)	225 (44.7)	
**Anxiety**													
No	3,693 (83.2)	3,608 (97.7)	85 (2.3)	0.093	3,333 (90.3)	360 (9.7)	<0.001	3,140 (85.0)	553 (15.0)	<0.001	2,624 (71.1)	1,069 (28.9)	<0.001
Yes	746 (16.8)	721 (96.6)	25 (3.4)		640 (85.8)	106 (14.2)		584 (78.3)	162 (21.7)		448 (60.1)	298 (39.9)	
**Depression**													
No	4,050 (91.2)	3,953 (97.6)	97 (2.4)	0.251	3,653 (90.2)	397 (9.8)	<0.001	3,398 (83.9)	652 (16.1)	0.961	2,785 (68.8)	1,265 (31.2)	0.041
Yes	389 (8.8)	376 (96.7)	13 (3.3)		320 (92.3)	69 (17.7)		326 (83.8)	63 (16.2)		287 (73.8)	102 (26.2)	
**Changes in sleep pattern**													
No	3,432 (77.3)	3,347 (97.5)	85 (2.5)	0.992	3,093 (90.1)	339 (9.9)	0.013	2,894 (84.3)	538 (15.7)	0.149	2,396 (69.8)	1,036 (30.2)	0.105
Yes	1,007 (22.7)	982 (97.5)	25 (2.5)		880 (87.4)	127 (12.6)		830 (82.4)	177 (17.6)		676 (67.1)	331 (32.9)	

Significantly more respondents with high (*p* = 0.014), moderate (*p* = 0.040) and low (*p* = 0.013) medical risks tested positive for COVID-19. Also, significantly more people not living with HIV than people living with HIV (PLHIV) had a positive SARS-CoV-2 test result (*p* < 0.001). In addition, significantly more people who had no fear of getting infected with COVID-19 (*p* < 0.001) and those who had the fear of infecting other persons (*p* < 0.001) tested COVID-19 positive.

Significantly more respondents who had COVID-19 symptoms but did not test were younger (*p* < 0.001), were males (*p* = 0.004), students (*p* = 0.002), had moderate (*p* < 0.001) and low (*p* < 0.001) health risks, had fear of getting infected (*p* < 0.001) and infecting someone else (*p* < 0.001), felt anxious (*p* < 0.001), depressed (*p* < 0.001) and had changes in sleep pattern (*p* = 0.013).

Significantly more respondents who had a friend who tested positive to COVID-19 were older (*p* = 0.002), males (*p* = 0.036), had college/university education (*p* < 0.001), were employed (*p* < 0.001), had moderate (*p* = 0.003) or low (*p* = 0.043) health risk, were not living with HIV (*p* < 0.001) and had the fear of infecting someone else (*p* < 0.001) and felt anxious (*p* < 0.001).

Significantly more respondents who knew someone who died of COVID-19 were older (*P* < 0.001), males (*p* < 0.001), had college/university education (*p* < 0.001), were retirees (*p* < 0.001), had mild, moderate or high health risk profiles (*p* < 0.001), were not living with HIV (*p* < 0.001), had the fear of getting infected (*p* = 0.001) or infecting others (*p* < 0.001), felt anxious (*p* < 0.001) and did not feel depressed (*p* = 0.041).

Table 2 highlights the factors associated with COVID-19 related fear, anxiety, depression and changes in sleep pattern. The *p*-values of the omnibus tests of model coefficients for the four models indicate that the models outperformed the null models. The goodness of fit tests also indicated that the models were robust except the model to determine the factors associated with depression.

**Table 2:** Logistic regression analysis for factors associated with anxiety, depression and sleep changes during the COVID-19 pandemic by adults in Nigeria (*N* = 4,439).

**Variables**	**Fear**		**Anxiety**		**Depression**		**Sleep changes**	
	**AOR (95% CI)**	***P*-value**	**AOR (95% CI)**	***P*-value**	**AOR (95% CI)**	***P*-value**	**AOR (95% CI)**	***P*-value**
Age	1.00 (0.99–1.01)	0.965	1.00 (0.99–1.01)	0.892	0.95 (0.94–0.96)	<0.001	0.99 (0.98–0.99)	<0.001
**Sex**								
Male (ref: Not male)	1.15 (1.01–1.30)	0.030	0.77 (0.65–0.91)	0.002	0.90 (0.71–1.14)	0.389	0.82 (0.71–0.95)	0.007
**Level of education**								
No formal education	1.00	–	1.00	–	1.00	–	1.00	–
Primary	0.80 (0.31–2.04)	0.634	0.98 (0.68–2.08)	0.960	0.30 (0.11–0.79)	0.015	0.99 (0.30–3.31)	0.984
Secondary	0.67 (0.30–1.46)	0.315	0.46 (0.24–0.87)	0.017	0.49 (0.23–1.05)	0.066	1.47 (0.55–3.90)	0.442
College/university	0.56 (0.26–1.22)	0.146	0.43 (0.23–0.80)	0.008	0.48 (0.23–1.02)	0.057	1.39 (0.53–3.66)	0.504
**Employment status**								
Employed (ref: Not employed)	1.25 (1.08–1.46)	0.003	1.18 (0.97–1.43)	0.109	0.82 (0.64–1.06)	0.134	0.79 (0.67–0.94)	0.008
**Health profile**								
High risk (ref: No high risk)	1.69 (1.17–2.45)	0.005	1.40 (0.97–2.03)	0.075	1.66 (1.03–2.69)	0.038	1.25 (0.86–1.50)	0.245
Moderate risk (ref: No moderate risk)	1.61 (1.34–1.93)	<0.001	2.61 (2.15–3.18)	<0.001	7.88 (6.14–10.10)	<0.001	1.57 (1.29–1.92)	<0.001
Low risk (ref: No low risk)	1.16 (0.94–1.44)	0.160	1.50 (1.18–1.90)	0.001	1.50 (1.09–2.07)	0.013	1.86 (1.50–2.32)	<0.001
**HIV status**								
Living with HIV (ref: Not living with HIV)	3.88 (3.22–4.69)	<0.001	1.64 (1.32–2.04)	<0.001	2.49 (1.89–3.28)	<0.001	0.30 (0.23–0.39)	<0.001
**COVID-19 status**								
**Tested COVID-19 positive**								
Yes (ref: No)	0.56 (0.37–0.85)	0.006	1.01 (0.62–1.65)	0.966	1.41 (0.73–2.72)	0.300	0.78 (0.49–1.26)	0.309
**Had COVID-19 symptoms but no test**								
Yes (ref: No)	1.61 (1.30–1.99)	<0.001	1.28 (0.99–1.64)	0.059	1.41 (1.02–1.94)	0.038	1.15 (0.92–1.45)	0.226
**Friend tested positive to COVID-19**								
Yes (ref: No)	1.28 (1.07–1.53)	0.008	1.35 (1.08–1.68)	0.007	1.06 (0.76–1.49)	0.726	1.06 (0.86–1.30)	0.579
**Knew someone who died of COVID-19**								
Yes (ref: No)	1.43 (1.24–1.65)	<0.001	1.53 (1.28–1.84)	<0.001	0.79 (0.60–1.04)	0.089	1.05 (0.89–1.24)	0.551
Nagelkerke *R*^2^	0.123		0.096		0.209		0.076	
Omnibus test of model coefficients	430.34	<0.001	261.12	<0.001	436.05	<0.001	227.29	<0.001
Hosmer and Lemeshow goodness of fit test	6.515	0.590	13.26	0.103	24.11	0.002	8.72	0.367

*AOR, adjusted odds ratio; CI, confidence interval*.

The factors associated with significantly higher odds of having COVID-19 related fear were being a male (AOR: 1.15; 95% CI: 1.01–1.30); being employed (AOR: 1.25; 95% CI: 1.08–1.46); having high (AOR: 1.69; 95% CI: 1.17–2.45) and moderate (AOR: 1.61; 95% CI: 1.34–1.93) health risk; living with HIV (AOR: 3.88; 95% CI: 3.22–4.69); having COVID-19 symptoms but not yet tested (AOR: 1.61; 95% CI: 1.30–1.99); having a friend who tested positive to COVID-19 (AOR: 1.28; 95% CI: 1.07–1.53) and knowing someone who died from COVID-19 (AOR: 1.43; 95% CI: 1.24–1.65). Having tested positive to COVID-19 was associated with significantly lower odds of experiencing fear (AOR: 0.56; 95% CI: 0.37–0.85).

Also, respondents had significantly higher odds of feeling anxious when they had moderate (AOR: 2.61; 95% CI: 2.15–3.18) or low (AOR: 1.50; 95% CI: 1.18–1.90) health risk profile; living with HIV (AOR: 1.64; 95% CI: 1.32–2.04); had a friend who tested positive for COVID-19 (AOR: 1.35; 95% CI: 1.08–1.68) or knew someone who died from COVID-19 (AOR: 1.53; 95% CI: 1.28–1.84). The odds of feeling anxious were significantly lower for respondents who were males (AOR: 0.77; 95% CI: 0.69–0.91); and those with secondary (AOR: 0.46; 95% CI: 0.24–0.87) or college/university (AOR: 0.43; 95% CI: 1.25–4.39) education when compared with those that had no formal education. Respondents who had significantly higher odds of feeling depressed had high (AOR: 1.66; 95% CI: 1.03–2.69), moderate (AOR: 7.88; 95% CI: 6.14–10.10) and low (AOR: 1.50; 95% CI: 1.09–2.07) health risks; living with HIV (AOR: 2.49; 95% CI: 1.89–3.28); and respondents who had COVID-19 symptoms but had not taken a test (AOR: 1.41; 95% CI: 1.02–1.94). The odds of feeling depressed were significantly lower for respondents who were older (AOR: 0.95; 95% CI: 0.94–0.96); and who had primary school education (AOR: 0.03; 95% CI: 0.11–0.79) when compared with those that had no formal education.

Factors associated with significantly higher odds of having sleep pattern changes were having moderate (AOR: 1.57; 95% CI: 1.29–1.92) or low (AOR: 1.86; 95% CI: 1.50–2.32) health risk profiles. Factors associated with significantly lower odds of having sleep pattern changes were being older (AOR: 0.99; 95% CI: 0.98–0.99); being a male (AOR: 0.82; 95% CI: 0.71–0.95); employed (AOR: 0.79; 95% CI: 0.67–0.94); and living with HIV (AOR: 0.30; 95% CI: 0.23–0.39).

## Discussion

The study identified COVID-19 related factors associated with the experience of fear, depression, anxiety and changes in sleep pattern during the pandemic. First, we identified that respondents who had COVID-19 symptoms but not yet tested, who had a friend who tested positive and who knew someone who died from COVID-19 had higher odds of being afraid while those who had tested positive to COVID-19 had lower odds of experiencing fear. Anxiety was higher for persons who had a friend who tested positive for COVID-19 and who knew someone who died from COVID-19. Those who had COVID-19 symptoms but had not taken a test had higher odds of being depressed. Second, respondents with low and moderate health risks had higher odds of feeling depressed, anxious or having changes in sleep pattern during the pandemic while those with moderate and high health risk profiles had higher odds of having fears (fear of contracting infection or infecting others). Third, PLHIV had higher odds of having fears, feeling anxious or depressed than people not living with HIV. They also had lower odds of changes in sleep patterns than people not living with HIV. Fourth, males had higher odds of having COVID-19 related fears, and lower odds of having anxiety and changes in sleep patterns; older respondents had lower odds of feeling depressed and having changes in sleep patterns; those with secondary or college/university education had lower odds of feeling anxious, while those with primary school education had lower odds of feeling depressed than respondents without formal education.

The study provides evidence that the experience of fear, depression, anxiety, and changes in sleep patterns differ between different populations. We observed that some populations that had higher odds of being afraid and higher odds of having being anxious (having moderate and low health risk for COVID-19, PLHIV, having a friend tested positive to COVID-19, knowing someone who died from COVID-19); higher odds of being depressed (having high, moderate and low health risk for COVID-19) and higher odds of having changes in sleep patterns (having moderate and low health risk for COVID-19). Others had higher odds of being afraid but lower odds of having anxiety (males) and changes in sleep patterns (being employed, PLHIV). The complex relationship between fear, anxiety, depression, and changes in sleep patterns was reflected in the results we report about PLHIV. PLHIV had higher odds of having fears and feeling anxious or depressed, but lower odds of changes in sleep patterns.

Also, our study findings that respondents who had COVID-19 symptoms but not yet tested, who had a friend who tested positive and who knew someone who died from COVID-19 was associated with higher odds for fear and anxiety is an indication for identifying individuals with this profile and providing psychological support to them. Their fears and anxiety may be related with concerns about they themselves likely testing COVID-19 positive, the stigma associated with this status (31) and the concerns with being quarantined (32). Their fears and anxiety may also be due to concerns with the attendant consequences of testing positive (2) such as facing stigma (33), boredom, frustration, inadequate supplies, inadequate information, and financial loss while in quarantine or isolation (2). Quarantine and isolation are also associated with anger, confusion, and post-traumatic stress symptoms (2). Positive public messaging about COVID-19 positive status may also go a long way to ameliorates these concerns about COVID-19 that triggers negative emotions.

These associations suggest that there may be various factors that mediate and/or moderate the relationship between fear, depression, anxiety and changes in sleep patterns. One of these factors may be age: we observed that respondents who are older had lower odds of feeling depressed or having changes in sleeping patterns. Aging is associated with an intrinsic reduction in susceptibility to depression (34) though people with chronic illness are more likely to be depressed (34–41) and have changes in sleep pattern due to physiological alterations (42, 43). People with high health risks are usually older (44–47). Our study findings indicated that those with high, moderate, and low health risk profiles had higher odds of reporting depression, anxiety and changes in sleep pattern corroborating prior findings (34–43). Populations with health concerns during the COVID-19 pandemic may however, have heightened concerns due to their susceptibility to infection and the absence of known therapies and vaccines. This may explain the high risk for depression, anxiety and changes in sleep pattern. On the other hand, this profile may have changed with the increased access to COVID-19 vaccines. The possibility of these changes may need to be explored in future studies.

Gender may act as a mediator and/or moderator of the relationship between fear, anxiety, and changes in sleep patterns. Though females were previously reported to be more likely to have fears (48), we observed in our study that males had higher odds of reporting fears. However, like a prior study, males had lower odds of reporting anxiety (49). We also observed that men had lower odds of changes in sleep patterns similar to prior studies that indicated that males had better sleep quality even during the pandemic (50, 51). This change in gender related association with fears during the pandemic may be related with men's concern about possible loss of income and the ability to provide the basic needs of the family. Although the International Labor Organization had stated that the pandemic had a greater impact on women than men in developed economies (52) this may not be the case for developing economies where men are responsible for securing food and life expenses and as such, may have greater concerns about losing their jobs due to COVID-19. Nigeria is a patriarchal society where men are the bread winners (53–55). With the loss of jobs and diminished income resulting from the pandemic (56–58), the affected male breadwinners may have fears. In the absence of welfare and social security packages during this pandemic for residents in Nigeria, there is a risk for an increase in health problems such as hypertension, high blood sugar and other metabolic disorders (59). This risk may be ameliorated by the lower risk for anxiety and sleep changes. This does not eliminate the possible need for palliative care for employees in Nigeria to absorb the economic shock they face because of the pandemic and reduce its impact on their quality of life.

Educational status is another possible mediator and/or moderator for anxiety and depression. Those with secondary education and above had lower odds of feeling anxious and those who had primary school education had lower odds of having depression than those without formal education. Prior studies indicated lower risk of depression and anxiety as the educational level improves (60, 61), while other evidence suggested no significant effect of educational level on anxiety (62). Like previous studies, we found that higher educational status was associated with lower odds of anxiety and depression during the pandemic. This finding may be because educated individuals may be more aware of modes of COVID-19 transmission and its consequences (63). Also, higher educational status may also be associated with better opportunities for employment, being male, lower risk for losing a job and thus, lower risk of experiencing anxiety and depression during the pandemic. This hypothesis needs to be tested further.

One of the strengths of this study is the large sample providing adequate study power. The data was also collected using validated tools and this strengthened the validity of the study findings. The data included information on the health status of respondents, which is relevant as differences in sickness status could influence anxiety, depression, and sleep pattern. The study has a few limitations despite its strengths. The self-reporting of fear, depression, anxiety, and HIV status is associated with high risk of social desirability and central tendency bias (64); and self-report may be more sensitive to identifying non-depressed, non-anxious and HIV negative individuals (65, 66). Also, we had an imbalance between participants on educational level, with comparably larger number of respondents with tertiary education which does not reflect the educational status of Nigeria. In addition, the study can only be generalized to those with internet access who could respond to the questionnaire; and it could not measure changes in the respondents' answers at different time points and phases of the pandemic as we know that the pandemic changed over time.

## Conclusion

Various factors were identified to be significantly associated with experiencing fear, anxiety, depression and change in sleep patterns among the participants during the pandemic. The study findings suggest that the pandemic may have had significant impact on the psychological wellbeing and daily living of individuals. Capacity building and training on how to deal and cope with stressful events and to enhance individuals' resilience are of paramount importance during large-scale crisis like the current pandemic. Besides, our study findings open avenues for further longitudinal assessment of the impact of COVID-19 pandemic on various life domains, considering the dynamic nature of the crisis and human behavior.

## Data Availability Statement

The raw data supporting the conclusions of this article will be made available by the authors, without undue reservation.

## Ethics Statement

The studies involving human participants were reviewed and approved by Research Ethics Committee at the Institute of Public Health of the Obafemi Awolowo University Ile-Ife, Nigeria. The patients/participants provided their written informed consent to participate in this study.

## Author Contributions

The project was conceptualized by MF. The data for the research was collected by MF, II, FL, and BP. The data analysis was conducted by OI. All authors contributed to the article, read, and approved the submitted version.

## Funding

AN was supported by funding from the National Institutes of Health/National Institute on Aging (K01 AG064986-01). All other authors made the needed personal financial contributions for the conduct of the study.

## Author Disclaimer

The contents of this paper do not necessarily represent the official views of the National Institutes of Health.

## Conflict of Interest

The authors declare that the research was conducted in the absence of any commercial or financial relationships that could be construed as a potential conflict of interest.

## Publisher's Note

All claims expressed in this article are solely those of the authors and do not necessarily represent those of their affiliated organizations, or those of the publisher, the editors and the reviewers. Any product that may be evaluated in this article, or claim that may be made by its manufacturer, is not guaranteed or endorsed by the publisher.

## Abbreviations

AOR: Adjusted Odds Ratio
CI: Confidence Interval
COVID-19: Corona Virus Infectious Disease – 2019
HIV: Human Immunodeficiency Virus
PTSD: Post Traumatic Stress Disorder
SARS-CoV-2: Severe Acute Respiratory Syndrome Corona Virus Type 2.
